# Research on path planning of robotic arms based on DAPF-RRT algorithm

**DOI:** 10.1371/journal.pone.0323734

**Published:** 2025-05-15

**Authors:** Zhenggang Wang, Junyang Tang, Fangxu Yi, Xiangrui Ren, Kunxiang Wang

**Affiliations:** School of Electrical Engineering, Anhui Polytechnic University, Wuhu, China; Tongji University, CHINA

## Abstract

In response to the widely used RRT-Connect path planning algorithm in the field of robotic arms, which has problems such as long search time, random node growth, multiple and unsmooth path turns, a path planning algorithm combining dynamic step size and artificial potential field is proposed. To solve the problem of scattered sampling points in the RRT-Connect algorithm, a goal-biased strategy is introduced. To address the problem of slow expansion caused by using fixed step sizes, a dynamic step size strategy is introduced to dynamically adjust the step size. To reduce randomness in the expansion process, the artificial potential field method is integrated to constrain the growth of new nodes by the random sampling function, the target gravitational function and the repulsion function. Finally, the planned path is pruned and smoothed using cubic B-splines to improve redundant points and turns in the path, and reduce the occurrence of shaking during the motion of the robotic arm. In the same environment, the improved algorithm reduces path length by 15.4% and planning time by 49.2%, compared with the RRT-Connect algorithm.

## 1 Introduction

Robotic arms, with their large workspace, ease of operation, and high flexibility [1], are gradually being adopted in key industries such as automobile manufacturing, shipbuilding, and aviation industry. At the same time, due to increasingly complex and variable operating environments, traditional methods such as manual teaching are gradually difficult to meet the requirements of efficient production. Against this backdrop, the ability to quickly and effectively plan a collision-free movement path for robotic arms has gained increasing attention and research focus, becoming a hot topic in the field of robotics and other related areas of intelligent systems [2].

Path planning methods can be generally divided into three categories: graph search-based algorithms, random sampling methods for path planning, and bionics-based path planning. The classical path search algorithms based on graph theory include Dijkstra’s algorithm [3], the A* algorithm [4,5], etc., while classic bionics-based path planning algorithms include genetic algorithms [6], ant colony algorithms [7,8], and particle swarm optimization algorithms [9], among others.

As the exploration space expands from two to three dimensions, the complexity of path planning computations increases exponentially, rendering the aforementioned algorithms unsuitable for solving the problems of path planning in the complex configuration spaces typical of robotic arms. In response to the high-dimensional spatial environments in which robotic arms operate, sampling-based path planning methods represented by the RRT (Rapidly-exploring Random Tree) algorithm [10] have gradually become a research focus. Due to its simplicity and efficiency, the RRT algorithm has been widely applied in path planning of robotic arms. However, the RRT algorithm also has some shortcomings. Although its high degree of randomness helps to avoid falling into the local optimal solution, the RRT algorithm also leads to the blindness of the search, and the planned path is often not the shortest or optimal. In recent years, many scholars have optimized the RRT algorithm, leading to a variety of RRT variants designed to speed up convergence and improve search efficiency.

Ding et al. [11] proposed the EP-RRT* algorithm, which first utilizes RRT-Connect to generate an initial feasible path. Then, the initial path is extended to create an expanded region, where iterative heuristic sampling is conducted within this region. The combination of the EP-RRT* algorithm with RRT-Connect enhances the search efficiency of the RRT* algorithm in specialized environments. Li et al. [12] proposed an improved RRT-Connect algorithm that employs an adaptive step size strategy and constructs four random trees from the starting point, endpoint, and fixed points to tackle the issues of slow expansion and hastened convergence. Experimental results show that the number of iterations and the number of path nodes of the improved algorithm are reduced by 18.11% and 23.02%, respectively. Chen et al. [13] proposed an enhanced RRT-Connect algorithm for the path planning of mobile robots. The algorithm generates a third node in space and concurrently constructs four trees for greedy expansion, in addition to implementing a goal-biased strategy to accelerate the search process. The findings demonstrate that the improved algorithm surpasses the RRT, RRT-Connect, and RRT* algorithms in terms of iteration times, planning time, and final path length under different environments. Liu et al. [14] proposed a bi-directional potential field probability step-size fast exploration random tree (BPFPS-RRT), in which a high-probability target bias strategy, and a step-size exploration strategy for target angles and random values are introduced. The improved algorithm has great improvement in search time and path length.

The DAPF-RRT (RRT algorithm with dynamic step size and artificial potential field) algorithm proposed in this paper innovatively introduces dynamic step size and artificial potential field on the basis of inheriting the advantages of the RRT algorithm. The dynamic step size strategy enables the algorithm to quickly expand the search space in the initial stage of search and finely adjust the step size when approaching the target, improving search efficiency and path accuracy. The artificial potential field guides the search direction, effectively avoiding falling into local optima and enhancing the global search ability of the algorithm in complex environments. Compared with existing methods, the DAPF-RRT algorithm can not only significantly shorten the path planning time but also generate smoother and better paths, showing stronger adaptability and robustness in complex environments and providing a more efficient and reliable solution for robot path planning.

## 2 Modeling and collision detection of robotic arms

### 2.1 Kinematics model of robotic arms

In this paper, the Mirobot [15], a six-axis desktop mini robotic arm is used for research.

**Fig 1:**
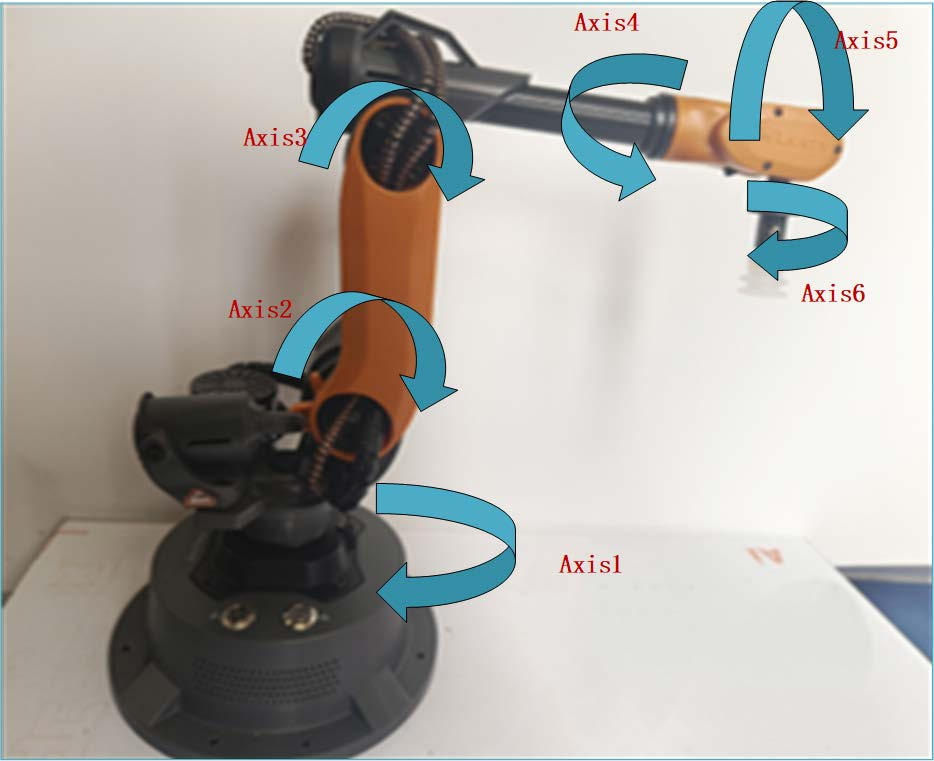
Mirobot six-axis robot arm.

**Fig 2:**
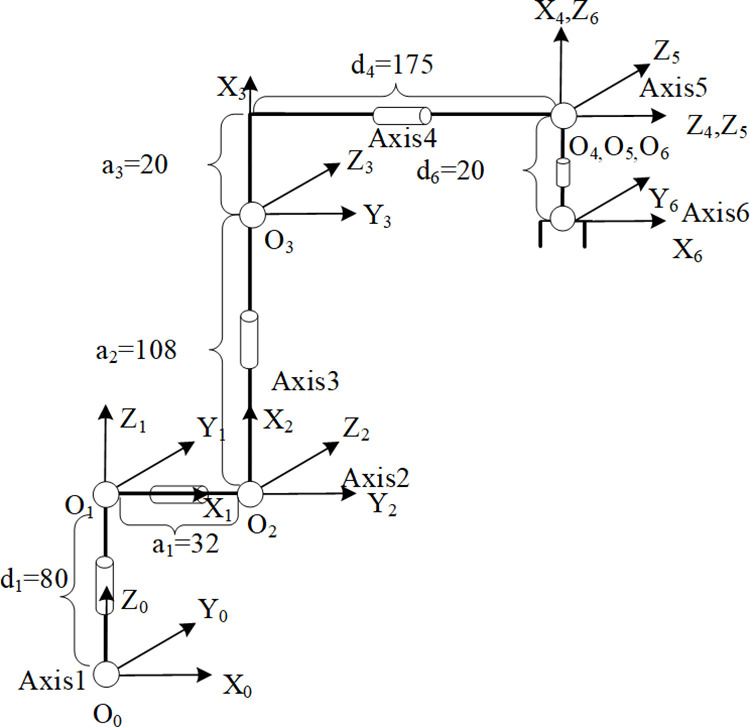
Joint coordinate system.

The shape and joints of the robotic arm are shown in Fig 1. The Denavit-Hartenberg (D-H) parameter method [16] is divided into the standard D-H parameter method and the modified D-H parameter method. In this paper, the modified D-H parameter method is used for modeling. The coordinate systems of each link are shown in Fig 2, and the modified D-H parameters are presented in Table 1.

**Table 1:** Modified D-H parameters.

i	${\alpha_{n}}_{-1}$	$a_{n-1}$	$d_{n}$	$\theta_{\text{n}}$
1	0	0	d_1_	$\theta_{1}$
2	-π/2	a_1_	0	$\theta_{2}$
3	0	a_2_	0	$\theta_{3}$
4	-π/2	a_3_	d_4_	$\theta_{4}$
5	π/2	0	0	$\theta_{5}$
6	π/2	0	d_6_	$\theta_{6}$

According to the establishment of the linkage coordinate system and the table of the modified D-H parameter, a homogeneous transformation matrix $\backslash {(}_{n}^{n-1}T$ representing the relative transformation relationship between adjacent linkages can be obtained, as shown in Eq. (1):


$$\backslash {(}_{n}^{n-1}T=\left[\begin{array}{cccc} \cos\theta_{n} & -\sin\theta_{n} & 0 & a_{n-1}\\ \sin\theta_{n}\cos\alpha_{n-1} & \cos\theta_{n}\sin\alpha_{n-1} & -\sin\alpha_{n-1} & -\sin\alpha_{n-1}d_{n}\\ \sin\theta_{n}\sin\alpha_{n-1} & \cos\theta_{n}\sin\alpha_{n-1} & \cos\alpha_{n-1} & \cos\alpha_{n-1}d_{n}\\ 0 & 0 & 0 & 1 \end{array}\right]$$ (1)

The formula contains four Denavit - Hartenberg (DH) parameters of the improved DH method: the joint angle *θ*_*n*_, the link offset *b*_*n*_, the link length *a*_*n-1*_, and the link twist angle *α*_*n-1*_.

Where *θ*_*n*_ is the rotation angle around the *z*_*n-1*_ axis, which determines the degree of rotation of the link in the direction of this axis, directly affecting the bending or twisting angle of the robot arm joint. For example, when the robot arm performs a grasping action, the change of the *θ*_*n*_ value of a joint will change the orientation of the end of the robot arm. *α*_*n-1*_ is the twist angle around the *x*_*n-1*_ axis, which is used to describe the relative twisting of two adjacent links in the vertical direction, which has an important impact on the overall spatial configuration and flexibility of the robotic. *α*_*n*_ is the distance from the *z*_*n-1*_ axis to the *z*_*n*_ axis along the *x*_*n-1*_ axis, which determines the length of each section of the robot arm, which in turn affects the working space range of the robot arm. For example, a larger *α*_*n*_ value will make the robot arm extend more in the corresponding direction. *d*_*n*_ is the distance from the *x*_*n-1*_ axis to the *x*_*n*_ axis along the *z*_*n-1*_ axis, which also affects the spatial structure and motion characteristics of the robot arm.

The homogeneous transformation matrix $\backslash {(}_{n}^{n-1}T$ is of great significance. It integrates the rotation and translation information into a matrix and comprehensively describes the relative transformation relationship between adjacent links. Through it, the motion state of each link can be accurately quantified, providing an indispensable basis for the subsequent calculation of the pose of the end of the robot arm.

Multiplying each transformation matrix of the above formula, the matrix of the end of the robot arm relative to the base of the robot arm can be obtained simultaneously as Eq. (2):


$$\backslash {(}_{6}^{0}T{=}_{1}^{0}T\cdot_{2}^{1}T\cdot_{3}^{2}T\cdot_{4}^{3}T\cdot_{5}^{4}T\cdot_{6}^{5}T=\left[\begin{array}{cccc} n_{x} & a_{x} & o_{x} & p_{x}\\ n_{y} & a_{y} & o_{y} & p_{y}\\ n_{z} & a_{z} & o_{z} & p_{z}\\ 0 & 0 & 0 & 1 \end{array}\right]$$ (2)

**Fig 3:**
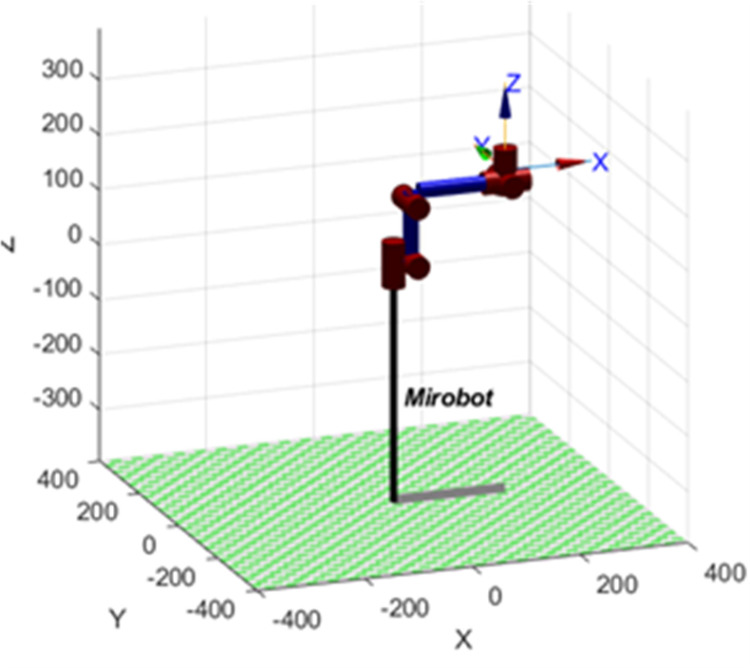
Mirobot robot arm model.

$\backslash {(}_{6}^{0}T$ represents the pose matrix of the end of the robot arm relative to the base. This matrix comprehensively reflects the final position and attitude information of the robot arm from the base to the end after various joint movements. Where *p* represents the position vector of the end of the robot arm, while *n*, *o* and *a* represent the vector coordinates of the end of the robot arm relative to its own coordinate system, respectively. D-H parameters are imported and Link functions and Serial link functions in Robotics Toolbox are used to build the model of the robotic arm. In this paper, the modified DH parameter method is used. The corresponding bars can be established by selecting “modified” and the initial joint position can be set by selecting “offset”. The established model of the robotic arm is shown in Fig 3.

In the control process of the robot arm, the transformation matrix is also crucial. When controlling the robot arm to perform tasks in real time, it is necessary to continuously obtain the actual pose information of the end of the robot arm and compare it with the desired pose. According to the pose deviation, the adjustment amount of each joint needs to be calculated through the transformation matrix, and then the control command is sent to the corresponding joint driver to realize the precise control of the movement of the robot arm.

### 2.2 Collision detection of robotic arms

Collision detection is an important part in the path planning of robotic arms, and it is also a prerequisite for planning a collision-free path. In the study by Xiong et al [17], it is emphasized that given the robotic arm’s composition of six parts, a more comprehensive discussion on its collision detection is necessary. This research offers in - depth exploration of collision - detection algorithms and strategies applicable to robotic arms, which could potentially inspire new ideas for handling the complex structure of our robotic arm during collision detection. For example, it might provide methods to better consider the movement ranges and interference possibilities of each of the six parts, as well as optimize the detection process for enhanced efficiency.

**Fig 4:**
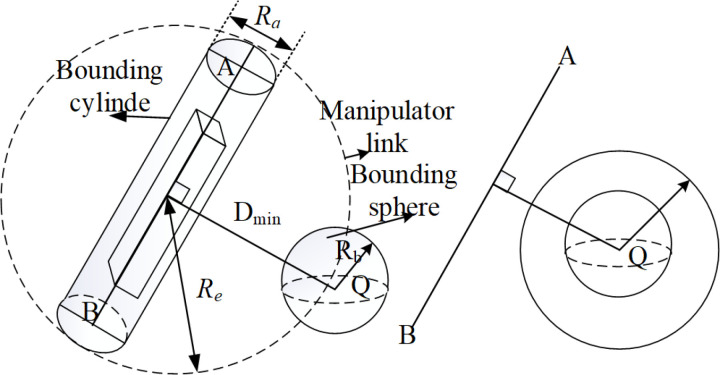
Schematic diagram of simplified model of the envelope method.

Furthermore, since both the robotic arm and obstacles are irregular objects, it is difficult to describe the interaction between them accurately, and challenging to simulate. Therefore, in order to improve the efficiency and accuracy of collision detection of the robotic arm in the workspace, the envelope method is used to simplify the collision detection between the robotic arm and obstacles. As shown in Fig 4(a), the robotic arm link is enveloped using a cylinder with radius Ra and the obstacle is enveloped using a sphere with radius Rb. As shown in Fig 4(b), through the simplification of the envelope method, the original collision detection between the robotic arm and the obstacle is transformed into the collision detection between the line segment and the sphere. By comparing the size of *D*_*min*_ and *Rc* and analyzing the position relationship between the AB line segment and the envelope sphere, it can be judged whether the robotic arm collides with the obstacle.

*R*_*a*_ intuitively reflects the actual radius size of the robot or obstacle in space and is an important basic dimension for judging the occurrence of a collision. When the distance between other objects and the surface of the cylinder is less than *R*_*a*_, a collision can be determined to occur. *R*_*e*_ is mainly used to give an early warning of collision risks. When other objects enter the circular area with a radius of *R*_*e*_, although an actual collision has not occurred yet, the collision detection algorithm will immediately enter the pre - detection stage. In this stage, the algorithm will more closely evaluate the possibility of the object colliding with the robot or obstacle at a future moment based on information such as the object’s motion trajectory and speed. Once the distance between the object and the center of the cylinder shrinks to be less than or equal to *R*_*a*_, a collision is determined to occur.

## 3 DAPF-RRT algorithm

### 3.1 Basic principles of RRT-Connect algorithm

**Fig 5:**
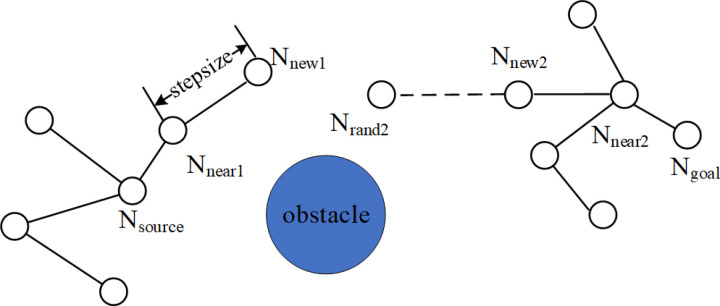
Schematic diagram of RRT-Connect algorithm expansion.

The RRT (Rapidly-exploring Random Tree) algorithm is a sampling-based path planning algorithm that is favored for its short planning time and strong exploration capabilities. However, it suffers from slow convergence speed. To improve the convergence rate of the algorithm, the RRT-Connect algorithm is proposed [18], and its expansion process is shown in Fig 5. The RRT-Connect algorithm enhances search efficiency and reduces search time through the following two key improvements:

(1) Unlike the RRT, which grows only one tree from the starting point, RRT-Connect constructs two trees, T1 and T2, simultaneously from the starting point and the end point. The two random trees grow close to each other until they are connected.
(2) After expanding the new node of the first tree T1 according to the RRT algorithm, it will try to expand the second tree T2 in the direction of the new node of T1, facilitating a rapid connection between the two trees. Furthermore, when the nodes of T2 can be expanded in this direction without encountering obstacles, it will continue to expand in this direction.

### 3.2 Path planning based on DAPF-RRT algorithm

The RRT-Connect algorithm uses two trees for bidirectional expansion and the speed is greatly improved compared with the RRT algorithm. However, the algorithm still faces problems such as scattered sampling points, random expansion, and low search efficiency due to the use of fixed step lengths, leading to paths with multiple turns. These problems remain to be solved.

#### 3.2.1 Goal bias.

In the RRT-Connect algorithm, target bias is added to make the nodes expand to the target point with a higher probability. This helps to address the problem of overly scattered sampling points and prevent node expansion from falling into dead zones. The idea of target bias is to set a sampling probability P∈[0,1] in the random sampling process. At the same time, it randomly generates a probability $P_{\text{rand}}$ between 0 and 1. Since the improved algorithm is a bidirectional search of two trees, if the probability $P_{\text{rand}}$ is less than the sampling probability, $N_{goal}$ or $N_{source}$ is used as the sampling point, otherwise the sampling point is randomly generated in the planning space by using Random() function. The sampling formula for adding goal bias is shown in Eq. (3). The setting of the threshold is related to the environment to be planned. When there are few obstacles in the environment, the threshold should be moderately increased to accelerate the expansion towards the target. When there are many obstacles in the environment, the threshold should be set smaller.


$$N_{rand}=\left\{ \begin{array}{c} {}*35l\;N_{goal}\;or\;N_{source},\;(P_{\text{rand}}<P)\\ \;\text{Random}(),\;(P_{\text{rand}}\geq P) \end{array}\right.$$ (3)

#### 3.2.2 Dynamically expanding the step size.

The RRT-Connect algorithm uses a fixed step size to expand new nodes for different environmental scenarios. For scenarios with simple environment, fixed step length does not significantly impact planning efficiency. However, in complex planning scenarios, when the tree expands into areas with dense obstacles, using a fixed step expansion increases the probability of collisions with obstacles. This leads to the failure of the new node expansions and initiates another search cycle, causing the algorithm spend more time bypassing obstacles. In this paper, combining with the step size calculation method proposed in reference [19], a dynamic step size strategy is introduced. The formula for calculating the step size is shown as Eq. (4):


$$\lambda_{d}=\left(\zeta {e^{A}}^{\cdot D_{obs}}-0.8\right)⬝\lambda$$ (4)

Where, λ is the initial step size, $\lambda_{d}$ is the adjusted dynamic step size, ζ is the adjustment coefficient, A is the attenuation coefficient, and $D_{obs}$ is the distance between *N*_*near*_ and the obstacle. When *N*_*near*_ is far from the obstacle, a larger step size is used for expansion to accelerate the convergence speed. When *N*_*near*_ approaches an obstacle, the step size changes with the decrease of $D_{obs}$, speeding up the algorithm to bypass the obstacle. By dynamically adjusting the step size, the strategy not only helps the tree to exit densely populated obstacle areas but also enhances the smoothness of the path. In the experimental process, the dynamic step size determined by the distance between the expanding random tree and the obstacles, is likely to be too small, leading to slow expansion within obstacle-dense areas. In this paper, the current dynamic expansion strategy is modified and the expression for the step size generated by the dynamic step size strategy is shown as Eq. (5):


$$\text{stepsize}=\left\{ \begin{array}{c} \lambda ,\;{D_{ob}}_{s}>D_{s};\\ \begin{array}{cc} & \;\lambda_{d},\;D_{obs}\leq D_{s}\\ & \;\lambda_{\min},\;\lambda_{d}<\lambda_{\min} \end{array} \end{array}\right.$$ (5)

**Fig 6:**
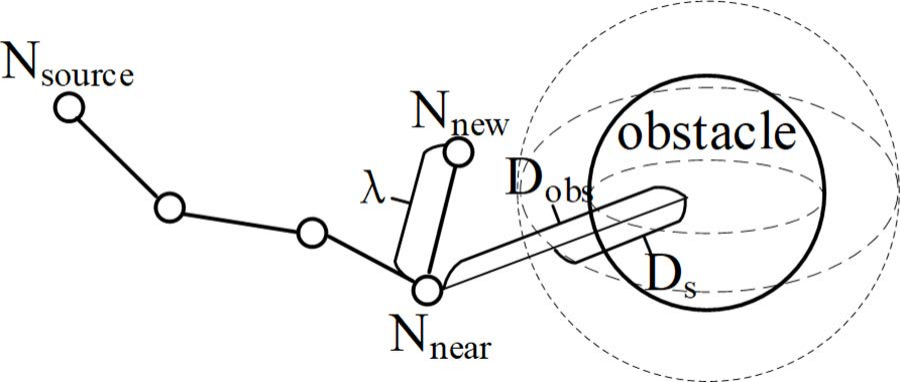
Schematic diagram of dynamic step size expansion.

As shown in Fig 6, step size adjustment range $D_{s}$ is first set. When the distance between *N*_*near*_ and the center of the obstacle ${D_{ob}}_{s}$ is greater than $D_{s}$, it means that its distance from the obstacle is far away, and the original step size is used for expansion. When the distance between *N*_*near*_ and the center of the obstacle ${D_{ob}}_{s}$ is smaller than $D_{s}$, the step size is dynamically adjusted. As ${D_{ob}}_{s}$ decreases, it means entering an obstacle-dense area, using a small step size to expand. When the step size is less than the minimum step size $\lambda_{\min}$, the minimum step size $\lambda_{\min}$ can be used to accelerate the exploration.

To clearly demonstrate the adaptive characteristics of the dynamic step - size strategy, two key parameters, *λ*_*d*_ and *λ*_*min*_, are involved in the figure. *λ*_*d*_ is the dynamic step - size adjustment coefficient. When a node is far from an obstacle, it increases the step size to accelerate convergence. When the node approaches the obstacle, *λ*_*d*_ decreases the step size to reduce the collision risk. *λ*_*min*_, is the minimum step size. If the dynamically adjusted step size is smaller than *λ*_*min*_, the algorithm uses *λ*_*min*_, for expansion to ensure exploration in complex obstacle - filled areas.

#### 3.2.3 Optimization of node expansion strategy.

In order to enhance the efficiency of the RRT-Connect algorithm in path planning, the randomness of expansions is minimized, and the convergence speed is accelerated. By integrating the target gravitational function and the repulsion function in the artificial potential field method into the growth process of the two trees, the node growth process is optimized. While overcoming the drawbacks of the traditional artificial potential field algorithm, which is prone to falling into local optima, it also makes better use of the information of obstacles in the map to improve the purpose of path search. The two trees can rapidly expand to the target point under the influence of the potential field.

The artificial potential field method has been widely used in the field of path planning due to its advantages of simple algorithm, small computational complexity, and fast convergence speed [20].

**Fig 7:**
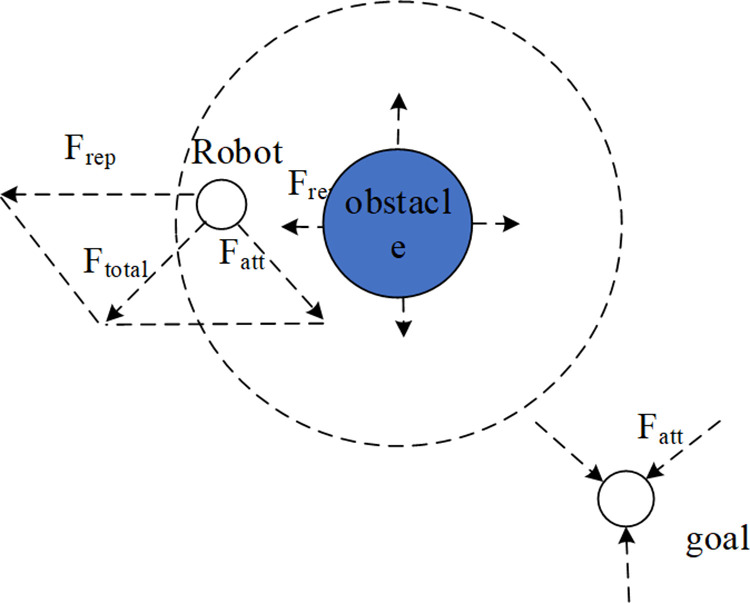
Artificial potential field method.

The basic principle of the artificial potential field is to set gravitational potential field at the target point and to create repulsion potential field by obstacles with a specified range of repulsion. Under the joint action of gravitational potential field and repulsion potential field, the robot moves towards the target along the direction of the total force and completes obstacle avoidance. The forces experienced by the robot within the artificial potential field are shown in Fig 7, where the dotted circle represents the range of the repulsive force from obstacles, F_att_ represents the attraction of the target point to the robot, F_rep_ represents the repulsive force from the obstacles, and F_total_ is the joint force of the two.

Drawing on the idea of the artificial potential field algorithm, the repulsion function and the target gravitational function are incorporated into the new node expansion of the RRT-Connect algorithm. The primary method is to set the gravitational field at the target point, which has a global influence. Each new node *N*_*new*_ that expands is constrained by the target gravitational function. At the same time, in order to prevent the target gravitation from being too strong and thus increase the probability of expansion failure in the obstacle-dense areas, the repulsion function is introduced. If *N*_*new*_ enters the range of the repulsion potential field, its growth will be affected by the direction of the resultant forces of the random sampling function, the target gravitational function and the repulsion function.

**Fig 8:**
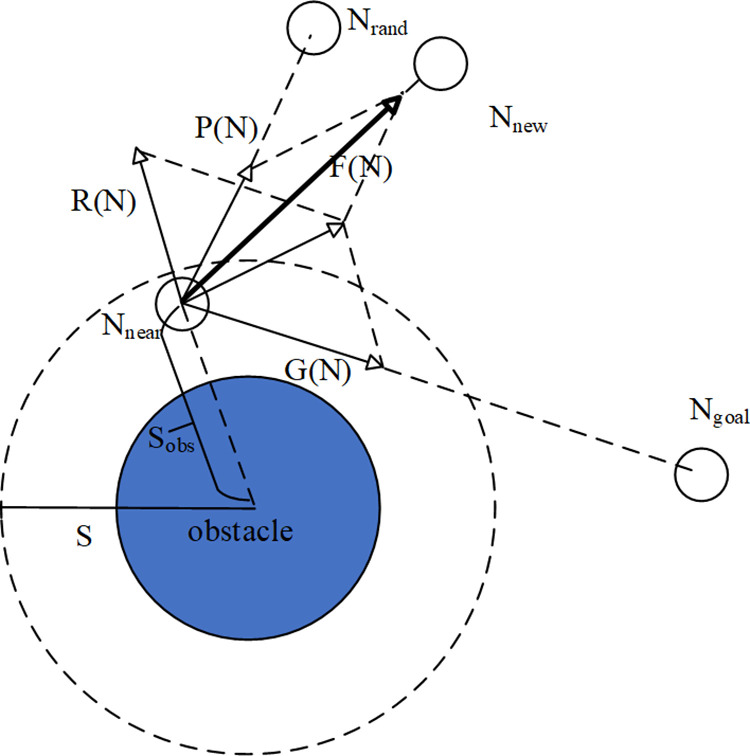
The target gravitational function and the repulsion function are added.

Fig 8 is a schematic diagram of the expansion method of new nodes after adding the target gravitational function and the repulsion function. In the diagram, R(N) represents the repulsion function, G(N) represents the target gravitational function and P(N) represents the random sampling function. F_total_(N) is the sum of G(N) and R(N). F(N) indicates the growth direction of the new node *N*_*new*._ S_obs_ represents the distance to the nearest obstacle from the nearest node *N*_*near*_, and S is the range of the repulsion force. Under the influence of the target gravitational function and the random sampling function, the new node is biased to expand towards the target point and the direction of expansion is adjusted by using the information of the surrounding obstacles. Each expansion brings the new node *N*_*new*_ closer to the target point, enhancing the purpose of the path search and significantly reducing the time required for path planning.

The unit vector of the target gravitational function $\vec{g}$ is shown in Eq (6), where $\left\|\begin{array}{c} N_{goal}-N_{near} \end{array}\right\|$ is the Euclidean distance between the two points *N*_*goal*_ and *N*_*near*_


$$\vec{g}=\frac{N_{\text{goal}}-N_{\text{near}}}{∥N_{\text{goal}}-N_{\text{near}}∥}$$ (6)

The target gravitational function G(n) can be constructed with Eq. (6), as shown in Eq. (7):


$$G(n)=\alpha \cdot \frac{N_{\text{goal}}-N_{\text{near}}}{∥N_{\text{goal}}-N_{\text{near}}∥}$$ (7)

Where, α is the gravitational coefficient of the target, and its value is related to obstacles in the space. When there are fewer obstacles in the space, α should be appropriately increased to speed up the expansion to the target. When there are many obstacles, α should be appropriately reduced to speed up the exploration to the barrier-free area.

Similarly, it can be concluded that the repulsion function of the node at *N*_*near*_ can be expressed as Eq. (8):


$$R(n)=\beta \cdot \frac{N_{\text{near}}-OBS}{∥N_{\text{near}}-OBS∥}$$ (8)

Where, *OBS* is the central coordinate of the obstacle, and β is the repulsion coefficient of the obstacle. The expression is shown in Eq. (9):


$$\beta =\left\{ \begin{array}{cc} {}*35l\frac{1}{2}K{\left(\frac{1}{S_{obs}}-\frac{1}{S}\right)}^{2}, & S_{obs}<S;\\ 0, & S_{obs}\geq S,S_{obs}<S_{\min} \end{array}\right.$$ (9)

Where, $S_{obs}$ is the distance from *N*_*near*_ to the nearest obstacle, S is the maximum range of the repulsion force of the obstacle, $S_{\min}$ is the minimum range of the repulsion force of the obstacle, and *K* is the gain coefficient. β increases with the decrease of $S_{obs}$ As $S_{obs}$ decreases, the repulsion function increases, which makes the node expand to the area far away from the obstacle. At the same time, in order to prevent the repulsion force from being too large, the minimum range of action $S_{\min}$ is set. When $S_{\min}$ is less than $S_{obs}$, the repulsion force is 0. The RRT-Connect algorithm is calculated by Eq. (10), where λ is the step size formula. After adding the target gravitational function and the repulsion function, the node calculation formula of DAPF-RRT algorithm is shown in Eq. (11), where $\lambda_{d}$ is the dynamic step size proposed in the previous section.


$$N_{new}=N_{near}+\lambda \cdot \frac{N_{rand}-N_{near}}{\left\|N_{rand}-x_{near}\right\|}$$ (10)

$$N_{new}=N_{near}+\lambda_{d}\cdot (\frac{N_{rand}-N_{near}}{\left\|N_{rand}-N_{near}\right\|}+R(n)+G(n))$$ (11)

#### 3.2.4 Path pruning.

**Fig 9:**
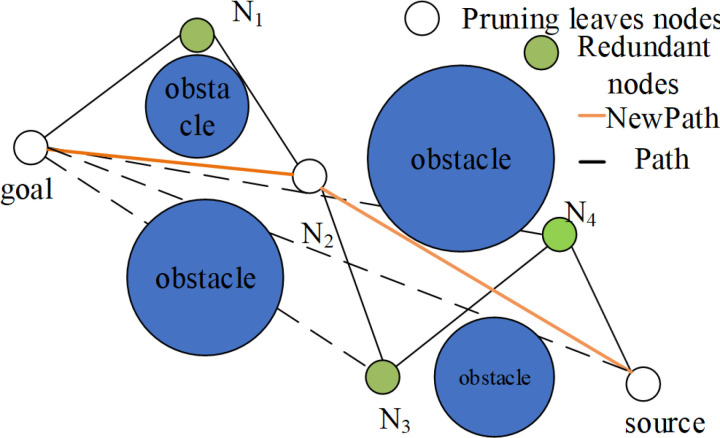
Schematic diagram of path pruning process.

After the aforementioned optimization of the RRT-Connect algorithm, the generated paths still contain many redundant nodes, especially in complex environments with numerous obstacles. To reduce unnecessary redundant nodes and improve the turning conditions in the path so that the path is closer to optimal, a greedy strategy is employed to prune the generated paths. The schematic diagram of the pruning process is shown in Fig 9, where the orange solid line represents the route after pruning.

The specific pruning steps are as follows:

(1) Create two variables, Path and New Path, to receive the path points before and after pruning. It is assumed that the final path is obtained by DAPF-RRT algorithm: Path= [N_source_, N_1_, N_2_, N_3_, ……N_q-2_, N_goal_];
(2) Take the target point *N*_*goal*_ as the starting point and try to connect all the nodes in the path in turn. The collision detection should be carried out for each connection. If there is a collision between *N*_*goal*_ and N_q_, the nodes between N_q-1_ and *N*_*goal*_ are all nodes that need to be pruned. Nq-1 is the node left after pruning. N_q-1_ is added to New Path;
(3) Take N_q-1_ as the starting point for the next pruning and repeat the above steps until the Path is traversed;
(4) Finally, according to the saved pruned nodes, the pruned path New Path=[N_source_, N_1_’, N_2_,’N_3_’, ……N_q-2_’, N_goal_] is obtained by backtracking.

#### 3.2.5 Path smoothing.

The path obtained by the DAPF-RRT algorithm is composed of continuous line segments sequentially connected by the expanded nodes of two random trees in space, which does not form a smooth curve. When a robotic arm moves along this unsmooth path, it may experience shaking, which can affect the stability of the robotic arm and increase wear and tear. To address this problem, it is necessary to perform path smoothing.

B-spline curves, known for their good continuity and locality, have been widely used in motion planning. Therefore, it is proposed to use B-spline curves to fit the pre-pruned path points to generate a smooth trajectory with continuous curvature. The expression for the *k-th* order B-spline curve is shown in Eq. (12).


$$C(u)=\sum_{i=0}^{n}d_{i}N_{i,k}(u)$$ (12)

In the equation, $d_{i}\left(i=0,\cdots ,n\right)$ is the control vertex coordinate, $u=[u_{0},u_{1},\cdots ,u_{n+k+1}]$ is the node vector of the B-spline function, and $N_{i,k}(0,\cdots ,n)$ is a *k*-degree B-spline basis function. The B-spline basis function is shown in the Eq. (13):


$$\left\{ \begin{array}{c} {}*35lN_{i,0}(u)=\left\{ \begin{array}{ccc} {}*35l1 & u_{i}\leq u\leq u_{i+1} & \\ & 0 & \text{other} \end{array}\right.\\ N_{i,k}(u)=\frac{u-u_{i}}{u_{i+k}-u_{i}}N_{i,k-1}(u)+\frac{u_{i+k+1}-u}{u_{i+k+1}-u_{i+1}}N_{i+1,k-1}(u) \end{array}\right.$$ (13)

Where, *i* is the node number and *k* is the order. The higher the order, the higher the smoothness, but the complexity of the calculation also increases. Considering the complexity of calculation and the requirement of path smoothing, the cubic B-spline curve is chosen and the fitting curve can meet the requirement of path smoothing in this paper. The cubic B-spline basis function is shown in Eq. (14):


$$\left\{ \begin{array}{c} {}*35lN_{0}(\text{u})=\frac{1}{6}(-u^{3}+3u^{2}-3u+1)\\ N_{1}(u)=\frac{1}{6}(3u^{3}-6u^{2}+4)\\ N_{2}(u)=\frac{1}{6}(-3u^{3}+3u^{2}-3u+1)\\ N_{3}(u)=\frac{1}{6}u^{3} \end{array}\right.u\in \left[0,1\right]$$ (14)

#### 3.2.6 DAPF-RRT algorithm flow.

**Fig 10:**
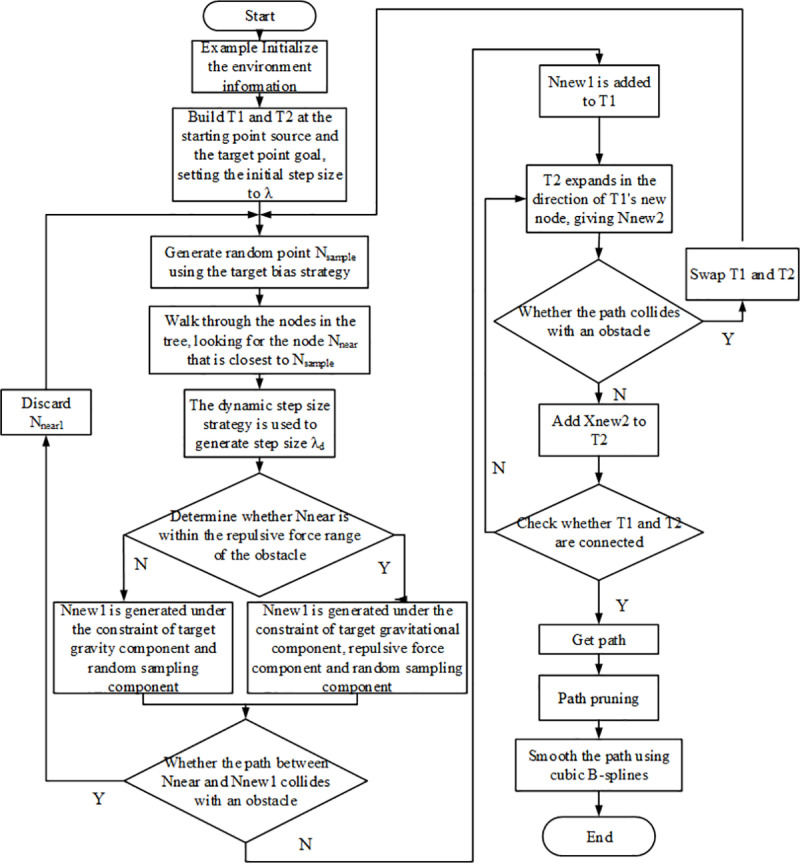
Flowchart of DAPF-RRT algorithm.

The overall flow of DAPF-RRT algorithm is shown in Fig 10. The specific steps of DAPF-RRT algorithm are as follows:

(1) Initialize the planned space and add obstacles. T1 and T2 are constructed at the starting point and the target point, and the initial step size is set as λ;
(2) Generate random points *N*_*sample*_ by using the target bias strategy, traverse the nodes in the tree, find the node *N*_*near*_ that is closest to *N*_*sample*_, and use the dynamic step strategy to generate step size $\lambda_{d}$;
(3) Determine whether *N*_*near*_ is within the repulsion range of the obstacles. If so, *N*_*new1*_ is generated under the constraints of the target gravitational function, the repulsion function and the random sampling function. Otherwise, *N*_*new1*_ is generated under the constraint of the target gravitational function and the random sampling function.
(4) Determine whether the path between *N*_*near*_ and *N*_*new1*_ collides with obstacles. If a collision occurs, *N*_*new1*_ will be discarded and return to step (2); otherwise, *N*_*new1*_ will be added to T1;
(5) T2 expands in the direction of new node *N*_*new1*_ of T1 to obtain *N*_*new2*_ and determine whether collision occurs on the generated path. If no collision occurs, *N*_*new2*_ is added to T2. If collision occurs, the random tree is exchanged and step (2) is returned;
(6) Determine whether T1 and T2 are connected, and return to the planned path point if they are connected. After the path is obtained, the path is pruned and smoothed using cubic B-splines.

The DAPF - RRT algorithm has a significant trade - off between computational complexity and path optimization performance.

In computational complexity, the dynamic step - size in the initial search uses large steps to cut sampling node calculations. Near the target, smaller steps boost accuracy but add local computation. The artificial potential field, while improving search efficiency, adds cost from force calculations. The traditional RRT’s complexity is about *O*(*mn*), (*m* is sampling points, *n* is search space dimension), and DAPF - RRT’s is around *O*(*mn* + *m*^*2*^), with *m*^*2*^ from potential field computations. For path optimization, the goal - bias strategy reduces sampling randomness, guiding nodes to the target. The dynamic step - size speeds up convergence far from obstacles and avoids collisions nearby. Though DAPF - RRT’s complexity rises due to these features, it improves path quality. In complex spaces, better paths need more computing. In an obstacle - filled area, the traditional RRT wastes time and gets long paths, while DAPF - RRT finds shorter paths faster, despite more calculations. As the environment gets more complex, DAPF - RRT’s complexity grows steadily and its path - finding ability gets better, achieving a good balance.

## 4 Three-dimensional space simulation experiment and analysis

To validate the feasibility and performance of the DAPF-RRT algorithm for path planning in complex three-dimensional spaces, an experiment was designed to compare the RRT, RRT-Connect and the DAPF-RRT algorithm under the same environmental conditions through simulation. In the experiment, the original RRT and RRT-Connect algorithms are modified to include target bias. The simulation environment is as follows. The operating system is Windows 10. The CPU is an Intel Core i5-1135G7 CPU @ 2.40 GHz, with 16GB of RAM. All simulation experiments are conducted using MATLAB 2021a software.

The search space set for the simulation experiment is [250 250 250], with a starting coordinate of [20 20 20] and an end coordinate of [200 200 180]. For the convenience of calculation, the obstacles in the experiment are spherical, and seven such spheres are set in the map. The respective coordinates of the center of the sphere are as follows: [125 125 125; 100 90 60; 150 100 80; 90 150 80; 200 200 120; 200 160 100; 40 180 100]. The respective radii are as follows: [30; 30; 20; 20; 20; 15; 20]. The initial step size is set to 10, and the maximum iterations times is set to 10000.

**Fig 11:**
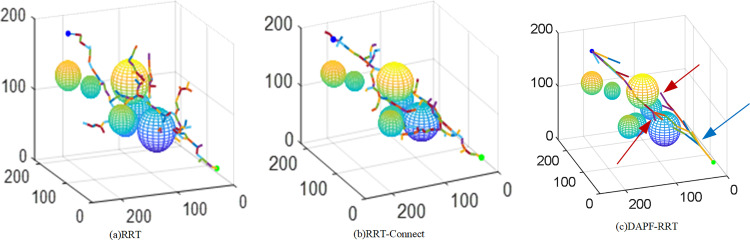
Expansion procedure.

The growth processes and final paths of the three RRT algorithms in the set three-dimensional space are shown in Fig 11. Fig 11(a) and 11(b) show the expansion process of the RRT and RRT-Connect algorithms, respectively. Due to the strong randomness of the two algorithms and the poor expansion orientation, a large number of ineffective branches are grown in the space during the expansion process.

Fig 11(c) shows the expansion process of the DAPF-RRT algorithm. It can be seen that the expansion is highly oriented and it grows rapidly from the two trees to the target point respectively. As shown by the blue arrow in Fig 11(c), the step size is larger at the position farther away from the obstacle during the expansion process, which accelerates the convergence speed. As shown by the red arrow in Fig 11(c), when expanding to an area with dense obstacles, the step size is smaller, which reduces the probability of collisions with obstacles, thereby finding a collision-free path more quickly.

**Fig 12:**
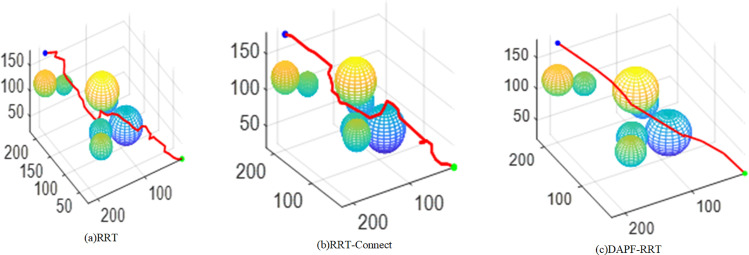
Final path.

The red line in Fig 12 represents the final planned path. From Fig 12(a) to 12(c), it is evident that the path planned by the improved algorithm is superior, with significantly fewer turns compared with the paths planned before the improvement.

**Fig 13:**
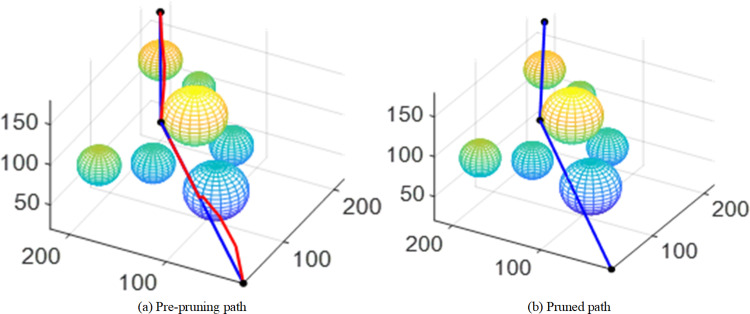
Comparison before and after path pruning.

The blue line in Fig 13 shows the path comparison after path pruning, with the red line in Fig 13(a) representing the path before pruning, and the blue line in Fig 13(a) representing the path after pruning. In Fig 13(b), the black dots on the blue line represent the optimal points retained after pruning. It can be clearly seen that the path after pruning is shorter, further reducing unnecessary turns and redundant nodes on the path.

**Fig 14:**
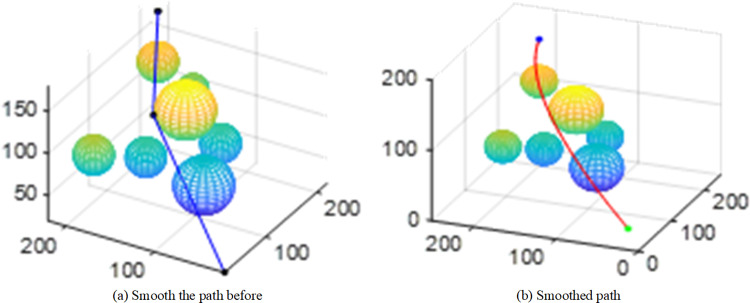
Path comparison before and after smoothing.

Cubic B-splines are used to smooth the pruned path, and the results are shown in Fig 14. The blue line in Fig 14(a) represents the path before smoothing and the red line in Fig 14(b) represents the path after smoothing. It can be seen from the figures that the pruned path becomes smoother at the turning point after cubic B-splines interpolation.

Due to the randomness of RRT series of algorithms, it is difficult to verify the superiority of the improved algorithm through one comparison. The DAPF-RRT algorithm and the other two algorithms are simulated for 30 times respectively in the environment of Fig 11. The average result of 30 times of path planning is shown in Table 2.

**Table 2:** Performance comparison of different algorithms.

Algorithm	Time/s	Path length	Nodes
RRT	8.1	390.5	40
RRT-Connect	6.7	372.2	38
DAPF-RRT	3.4	314.6	6

Compared with the RRT and RRT-Connect algorithms, the path length of the improved algorithm is reduced by 19.4% and 15.4% respectively. Compared with the other two algorithms, the time taken to plan a feasible path in space is reduced by 58% and 49.2% respectively. The average number of pruned nodes is approximately 6, which is 84.2% lower than that of RRT-Connect algorithm.

**Fig 15:**
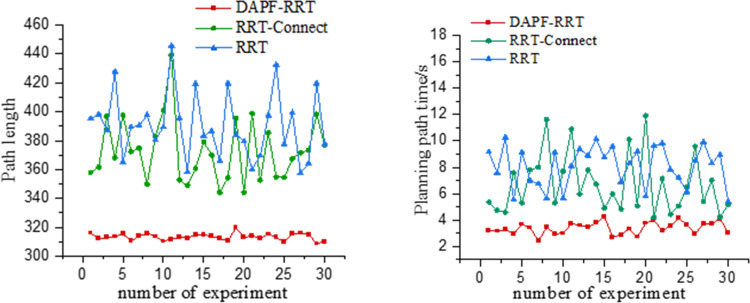
Time and path length of 30 simulation planning.

As shown in Fig 15, the performance of the improved algorithm has been greatly improved compared with the original algorithm, with significant improvements in planning time and path length.

To verify the effectiveness of the DAPF-RRT algorithm in different maps, 30 simulations are conducted on four maps with varying levels of complexity. The results are shown in Table 3. In Map1, the path length and planning time of the DAPF-RRT algorithm proposed in this paper are reduced by 18.3% and 48.8% respectively, compared with those before the improvement. In Map2, the path length and planning time of the DAPF-RRT algorithm are reduced by 16.9% and 43% respectively compared with the RRT-Connect algorithm.

**Table 3:** Path length and planning time of map planning with different complexity.

Algorithm	Map	Path length	Time/s
	Map1	307.7	2.2
DAPF-RRT	Map2	317.4	3.7
	Map3	320.4	4.1
	Map4	324.8	5.1
	Map1	376.5	4.3
RRT-Connect	Map2	381.8	6.5
	Map3	390.1	6.8
	Map4	417.9	8.8

In Map3, the path length and planning time of the algorithm proposed in this paper are reduced by 17.9% and 39.7% respectively compared with the RRT-Connect algorithm. In Map4, the path length and planning time of the algorithm proposed in this paper are reduced by 22.3% and 42% respectively compared with the RRT-Connect algorithm.

**Fig 16:**
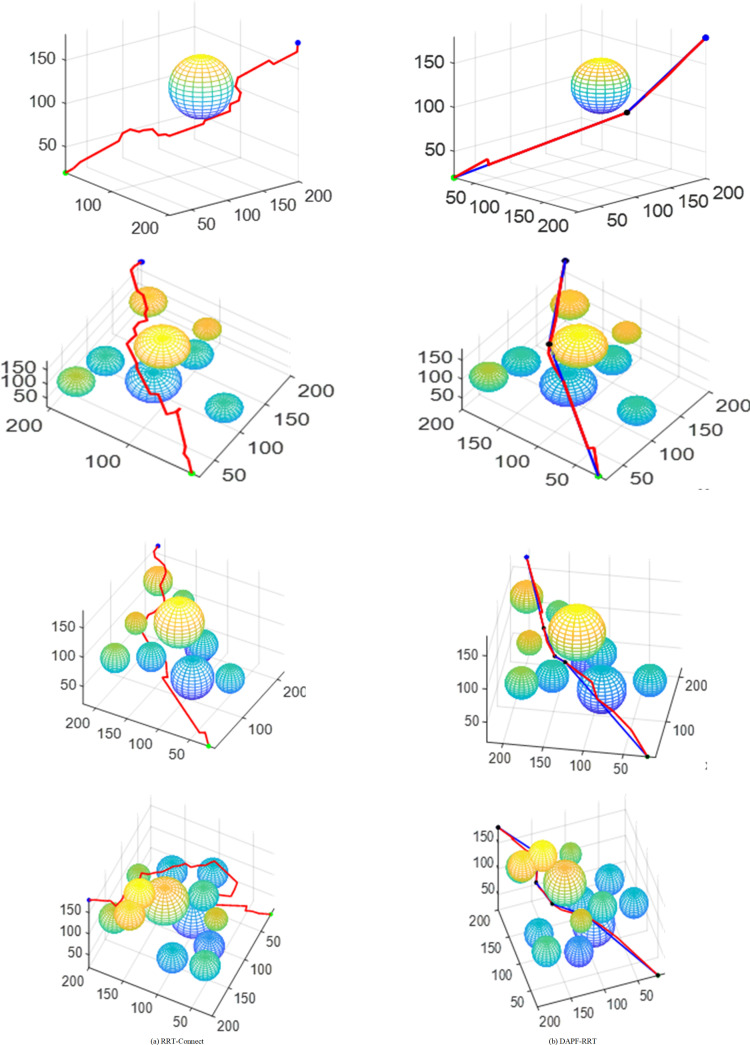
Map simulation comparison of different complexity.

Fig 16 shows the paths planned by the algorithms before and after the improvements in maps with varying levels of complexity. The red line in Fig 16(a) represents the path planned by the RRT-Connect algorithm while the path planned by the DAPF-RRT algorithm are shown in Fig 16(b), with the red line indicating the planned path and the blue line indicating the path after pruning. In three-dimensional spaces with varying complexity, the DAPF-RRT algorithm has shown better obstacle-avoidance effect and better path planning capabilities than the original algorithm.

## 5. Simulation for path planning of robotic arms

To further verify whether the paths planned in space by the DAPF-RRT algorithm proposed in this paper meet the kinematic constraints of the robotic arm after pruning and cubic B-spline smoothing, a six-axis robotic arm model is imported into MATLAB using the Robotics Toolbox and spherical obstacles are created in space, thus the path planning simulation is carried out using the DAPF-RRT algorithm.

**Fig 17:**
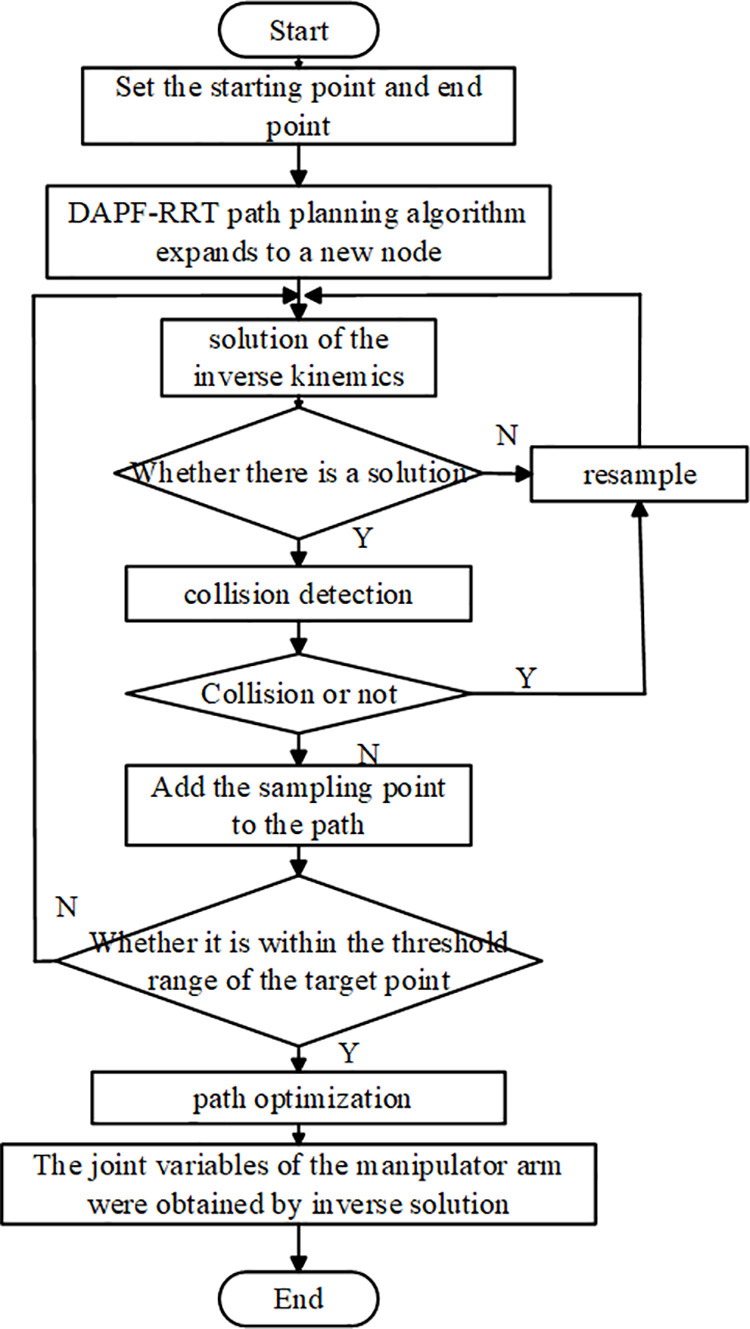
Path planning simulation process of the robotic arm.

The simulation process of the path planning of robotic arms is shown in Fig 17.

The specific steps of the simulation process are as follows:

(1) Set the starting and end points to initialize the three-dimensional space.
(2) Use the DAPF-RRT algorithm to generate a new candidate node.
(3) Based on the forward kinematic equation of the robotic arm, determine whether there is an analytical solution for the end - effector pose corresponding to the candidate node. If the solution obtained from the equation does not meet the motion limitations of the robotic arm, there is no analytical solution. Discard the node and adjust the sampling strategy as appropriate; if there is a solution, retain the node for subsequent detection.
(4) Use the envelope method to determine whether any of the links will collide with an obstacle at the node. If there is a collision, the node will be discarded. Otherwise, it will be considered as a potential path point.
(5) Determine whether the new node has reached the vicinity of the target area. If not, continue looping step (2). If it has reached the area, generate a preliminary path and proceed with subsequent optimization.
(6) For the path points of the preliminary path, solve the joint angles using the inverse kinematic model based on D - H parameters and an iterative algorithm. When there are multiple solutions, select the solution with smooth joint motion and low energy consumption. When there is no solution, first fine - tune the path point. If there is still no solution, backtrack and re - plan the path to ensure the feasible movement of the robotic arm.

**Fig 18:**
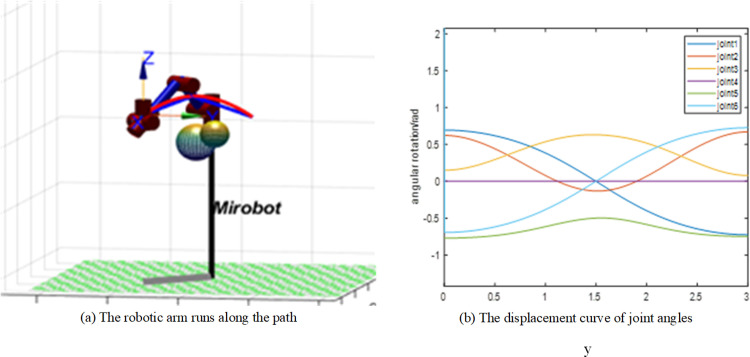
Effect diagram of robotic arm moving along trajectory.

The movement of the robotic arm along the path is shown in Fig 18(a). Fig 18(b) shows the joint angle displacement curve. The smoothed path can ensure the smooth operation of the robotic arm.

The operation of the robotic arm along the path is shown in Fig 18(a). The blue line in Fig 18(a) represents the path after pruning but before smoothing, and the red line represents the path after cubic B-spline smoothing. Fig 18(b) shows the displacement curves of the joint angles outputted after the movement of the robotic arm. When the robotic arm moves along the smoothed path, the displacement curves of the joint angles are continuous and free of abrupt changes, ensuring smooth operation of the robotic arm along the planned path.

## 6 Summary

The following improvements have been made to address the shortcomings of the RRT-Connect algorithm: goal-biased strategy is introduced to make new nodes expand toward the goal with a greater probability. To address the problem of slow expansion caused by the original algorithm’s use of a fixed step size, a dynamic step size has been introduced. By integrating the target gravitational function, repulsion function, and random sampling function to collectively constrain the expansion of new nodes, the node growth strategy is optimized, solving the problem of random node expansion and weak orientation in the RRT-Connect algorithm. A greedy strategy is used to prune the path after planning, making the path shorter and reducing the number of turns. The pruned path is made smoother using a cubic B-spline curve method, which reduces the shaking caused by frequent turns in the path when the robotic arm moves along it. The effectiveness of the DAPF-RRT algorithm has been validated through MATLAB, and path planning for the robotic arm was simulated using the Robotics Toolbox in MATLAB. The simulation shows that the displacement curves of joint angles of the robotic arm are quite smooth.

Looking ahead, the DAPF - RRT algorithm holds extensive prospects and substantial research potential in multi - robot systems and dynamic obstacle environments. In the context of multi - robot systems, it is feasible to explore distributed implementation methods. Each robot operates the algorithm using local information and shares data, thereby avoiding collisions and enhancing efficiency, similar to its applications in the logistics field. Additionally, it is of great significance to conduct joint optimization of task allocation and path planning by integrating robot capabilities and task requirements. Regarding dynamic obstacle environments, integrating real - time sensor data to adjust the repulsive force function is crucial. This facilitates rapid adaptation to environmental changes and the replanning of safe paths, similar to real - time obstacle avoidance in autonomous driving. Meanwhile, enhancing the real - time replanning ability by adopting an incremental search strategy can shorten the replanning time and ensure the safe and efficient operation of robots in dynamic environments.

## References

[1] Kim SH, Nam E, Ha TI, Hwang S-H, Lee JH, Park S-H, et al. Robotic machining: a review of recent progress. Int J Precis Eng Manuf. 2019;20(9):1629–42. doi: 10.1007/s12541-019-00187-w

[2] Xu M, David JM, Kim SH. The fourth industrial revolution: opportunities and challenges. IJFR. 2018;9(2):90. doi: 10.5430/ijfr.v9n2p90

[3] Dijkstra EW. A note on two problems in connexion with graphs[M]. Edsger Wybe Dijkstra: His Life, Work, and Legacy. 2022. p. 287–90.

[4] Fransen K, van Eekelen J. Efficient path planning for automated guided vehicles using A* (Astar) algorithm incorporating turning costs in search heuristic. Int J Prod Res. 2021;61(3):707–25. doi: 10.1080/00207543.2021.2015806

[5] Zhong X, Tian J, Hu H, Peng X. Hybrid path planning based on safe A* algorithm and adaptive window approach for mobile robot in large-scale dynamic environment. J Intell Robot Syst. 2020;99(1):65–77. doi: 10.1007/s10846-019-01112-z

[6] Li Y, Zhao J, Chen Z, Xiong G, Liu S. A robot path planning method based on improved genetic algorithm and improved dynamic window approach. Sustainability. 2023;15(5):4656. doi: 10.3390/su15054656

[7] Ni Y, Zhuo Q, Li N, Yu K, He M, Gao X. Characteristics and optimization strategies of A* algorithm and ant colony optimization in global path planning algorithm. Int J Patt Recogn Artif Intell. 2023;37(03). doi: 10.1142/s0218001423510060

[8] Yang H, Qi J, Miao Y, Sun H, Li J. A new robot navigation algorithm based on a double-layer ant algorithm and trajectory optimization. IEEE Trans Ind Electron. 2019;66(11):8557–66. doi: 10.1109/tie.2018.2886798

[9] Huang C, Zhou X, Ran X, Wang J, Chen H, Deng W. Adaptive cylinder vector particle swarm optimization with differential evolution for UAV path planning. Eng Appl Artif Intell. 2023;121:105942. doi: 10.1016/j.engappai.2023.105942

[10] Veras LGDO, Medeiros FLL, Guimaraes LNF. Systematic literature review of sampling process in rapidly-exploring random trees. IEEE Access. 2019;7:50933–53. doi: 10.1109/access.2019.2908100

[11] Ding J, Zhou Y, Huang X, Song K, Lu S, Wang L. An improved RRT* algorithm for robot path planning based on path expansion heuristic sampling. J Comput Sci. 2023;67:101937. doi: 10.1016/j.jocs.2022.101937

[12] Li J, Huang C, Pan M. Path-planning algorithms for self-driving vehicles based on improved RRT-connect. Transp Saf Environ. 5(3):tdac061.

[13] Chen J, Zhao Y, Xu X. Improved RRT-connect based path planning algorithm for mobile robots. IEEE Access. 2021;9:145988–99. doi: 10.1109/access.2021.3123622

[14] Liu Y, Tao W, Li S, Li Y, Wang Q. A Path planning method with a bidirectional potential field probabilistic step size RRT for a dual manipulator. Sensors (Basel). 2023;23(11):5172. doi: 10.3390/s23115172 37299899 PMC10255475

[15] Zhou D, Xie M, Xuan P, Jia R. A teaching method for the theory and application of robot kinematics based on MATLAB and V‐REP. Comp Appl Eng Educ. 2019;28(2):239–53. doi: 10.1002/cae.22188

[16] Denavit J, Hartenberg RS. A kinematic notation for lower-pair mechanisms based on matrices. J Appl Mech. 1955;22(2):215–21. doi: 10.1115/1.4011045

[17] Xiong Y, Wei Q, Yang Z, Li P, Cheng H. Collision - free path planning for dual - arm robots based on improved RRT. In the 2024 IEEE 7th Advanced Information Technology, Electronics and Automation Control Conference (IAEAC). Chongqing, China; 2024. p. 433–9.

[18] Zhang L, Shi X, Yi Y, Tang L, Peng J, Zou J. Mobile robot path planning algorithm based on RRT_connect. Electronics. 2023;12(11):2456. doi: 10.3390/electronics12112456

[19] Cheng H, Yang S, Qi X. Dynamic trajectory planning of quadrotors UAV based on improved RRT algorithm. Comput Eng Des. 2018;39(12):3705–11.

[20] Zhang W, Wang N, Wu W. A hybrid path planning algorithm considering AUV dynamic constraints based on improved A* algorithm and APF algorithm. Ocean Eng. 2023;285:115333. doi: 10.1016/j.oceaneng.2023.115333

